# Electroacupuncture and manual acupuncture at LR3 and ST36 have attenuating effects on hypertension and subsequent cognitive dysfunction in spontaneously hypertensive rats: A preliminary resting-state functional magnetic resonance imaging study

**DOI:** 10.3389/fnins.2023.1129688

**Published:** 2023-03-09

**Authors:** Ji-peng Liu, Yin-yin Li, Ke-zhen Yang, Shu-feng Shi, Yu Gong, Zhuang Tao, Yi Tong, Jiao Sun, Bing-nan Yue, Xiao-lu Li, Xin-yu Gao, Qing-guo Liu, Meng Xu

**Affiliations:** ^1^School of Acupuncture-Moxibustion and Tuina, Beijing University of Chinese Medicine, Beijing, China; ^2^Department of Tuina, Beijing University of Chinese Medicine Third Affiliated Hospital, Beijing, China; ^3^Encephalopathy Center, The First Affiliated Hospital of Anhui University of Chinese Medicine, Hefei, China; ^4^Beijing Tong Ren Tang International Natural-Pharm Co., Ltd., Beijing, China

**Keywords:** spontaneously hypertensive rats, cognitive functions, electroacupuncture, manual acupuncture, rs-fMRI

## Abstract

**Introduction:**

Chronic hypertension may have a contributory role toward cognitive impairment. Acupuncture exerts protective effects on cognitive functions while controlling the blood pressure. However, the neural mechanism underlying the dual attenuating effect of acupuncture remains unclear. In this study, we investigated the effects of electroacupuncture (EA) and manual acupuncture (MA) on the functional activity of the brain regions of spontaneously hypertensive rats (SHRs) by through resting-state functional magnetic resonance imaging (rs-fMRI). We also evaluated the differences in these functional activities between the EA and MA groups.

**Methods:**

We randomly assigned 30 SHRs into the EA, MA, and model (SHR) groups. Wistar Kyoto rats (*n* = 10) were used as normal control (WKY). The interventions were administered once every alternate day for 12 weeks. The systolic blood pressure of all rats was recorded every 2 weeks until the end of the intervention. After the intervention, rs-fMRI scanning was performed to access the whole brain data of rats randomly selected from each group evenly. The amplitude of low frequency fluctuation (ALFF) analysis, regional homogeneity (ReHo) analysis, and functional connectivity (FC) analysis were also conducted. The Morris water maze (MWM) test was conducted to evaluate the learning and memory of the rats. Hematoxylin-eosin staining and Nissl staining were performed to observe histopathological changes in the key brain regions.

**Results:**

We demonstrated that, when compared with the SHR group, the EA and MA groups had significantly lower blood pressure and better performance for behavioral test indices, and that the effect of EA was better than that of MA. ALFF and ReHo analyses revealed enhancement of the neuronal activity of some functionally impaired brain areas in the EA and MA groups. The main callback brain regions included the hypothalamus, entorhinal cortex, brain stem, prelimbic cortex, cingulate cortex, corpus callosum, and cerebellum. The FC analysis demonstrated that EA and MA enhanced the functional connectivity between the seeds and brain regions such as the brain stem, entorhinal cortex, hippocampus, prelimbic cortex, and cerebellum. The pathological test of the entorhinal cortex also verified the protective effect of acupuncture on the neuronal functional activity.

**Discussion:**

Our findings suggested that EA and MA exhibited attenuating effects on hypertension and cognitive dysfunction by enhancing the functional activities in the corresponding brain regions. Moreover, EA activated more callback brain regions and functional connectivity than MA, which may explain why the effect of EA was better than that of MA.

## 1. Introduction

Hypertension, a common chronic disease prevalent worldwide, has been reported as one of the main risk factors for cardiovascular and cerebrovascular conditions such as coronary heart disease, stroke, and cognitive impairment (GBD 2015 Risk Factors Collaborators, 2016), and its prevalence keeps growing (Mills et al., 2020). Globally, the total number of adult patients with hypertension is estimated to increase to 1.56 billion by 2025 (Kearney et al., 2005), implying a severe challenge to public health. Past studies have reported that chronic hypertension may induce cognitive impairment or even cognitive dysfunction (Gottesman et al., 2014; Mills et al., 2016; Iadecola and Gottesman, 2019), mainly affecting learning and memory, attention, and executive function (Xue et al., 2019). Chronic hypertension also plays a contributory role toward the increase in the morbidity rate of vascular dementia and Alzheimer’s disease (Carnevale et al., 2020; García-Alberca et al., 2020), thereby bringing a huge economic burden to families and society. Therefore, investigating the pathogenesis and prevention of cognitive impairment followed by chronic hypertension is of great social significance.

Chronic hypertension may also damage the structural and functional integrity of brain microcirculation, leading to cerebral microvascular obstruction, impaired neurovascular coupling, and damage to the cerebral blood supply. In addition, hypertension may induce conditions associated with the occurrence of cognitive impairment, such as blood–brain barrier disruption, neuroinflammation, amyloid lesions, and white matter lesions (Ungvari et al., 2021). Although the pathogenesis of cognitive impairment followed by hypertension remains unclear, past studies have substantiated that these conditions can be prevented and treated (Walker et al., 2017; van Eersel et al., 2019).

Acupuncture, a type of complementary and alternative medicine that originated in China, has been incorporated as a complementary therapy in the clinical practice of hypertension (Chen et al., 2018). Past studies by our team (Wu et al., 2021; Yang et al., 2022a,b) have confirmed that acupuncture controls blood pressure in hypertensive patients and spontaneously hypertensive rats (SHRs) exhibiting an ameliorating effect on hypertensive target organs such as the brain, heart, and kidneys. Other studies (Lanari et al., 2007; Lai et al., 2012; Kim et al., 2013) have reported that acupuncture enhanced the recovery of damaged brain tissues by inhibiting neuronal apoptosis, enhancing neuronal plasticity, improving the cerebral blood flow, and regulating the brain metabolism. Although the action mechanism remains unclear, these studies have indicated that acupuncture may attenuate cognitive impairment followed by hypertension.

Resting-state functional magnetic resonance imaging (rs-fMRI) presents functional information about the brain according to the changes in the blood flow and oxygen cooperation in different brain regions in the resting state (Yousaf et al., 2018). In recent years, rs-fMRI has gradually been adopted in acupuncture studies for assessing acupuncture-induced changes in the brain activity signals, which are of great significance to further reveal the neuroimaging mechanism of acupuncture effect (Zhang et al., 2013; Wang et al., 2016). The commonly used rs-fMRI analyses include the amplitude of low-frequency fluctuation (ALFF), regional homogeneity (ReHo), and functional connectivity (FC) analyses. ALFF and ReHo analyses can directly reflect neuron spontaneous activity without the influence of model-generated errors (Liu et al., 2014). Specifically, ALFF analysis reflects the spontaneous activity level of each voxel from the perspective of energy (Liang et al., 2014), while ReHo analysis evaluates the synchronization and coordination of neuronal activities in the local brain regions in time (Liu et al., 2008). Increased ALFF and ReHo values indicate neuronal activation in the corresponding brain areas (Li et al., 2021). FC analysis reflects the functional connectivity between different brain regions (Hutchison et al., 2013). By conducting ALFF and ReHo analyses, some studies have reported that acupuncture exerts an antihypertensive effect to an extent by activating the blood pressure-regulating brain regions (Zhang et al., 2019, 2021). Furthermore, through FC analysis and using the key brain areas for blood pressure regulation as seeds, Zheng et al. (2016) found that acupuncture enhanced the functional connectivity between the hypothalamus and blood pressure-regulating brain regions.

Electroacupuncture (EA) and manual acupuncture (MA) are different in various aspects, such as action mechanisms, targets, and clinical efficacy (Stener-Victorin et al., 2006; Zhao, 2008; Johansson et al., 2013). Based on our understanding, only a few studies have adopted rs-fMRI in their analysis of the protective effects of EA and MA on the cognitive functions in the hypertensive model. Here, we selected SHRs to evaluate the attenuative effect of acupuncture methods on hypertension and cognitive impairment through the blood pressure analysis and the Morris water maze (MWM) test. rs-fMRI was performed for analyzing brain signal changes in SHRs and for comparing the brain functional activity between the EA and MA groups. We identified the neuroimaging mechanisms of EA and MA that affected blood pressure control and cognitive function protection in SHRs, thereby providing insights to identifying the target brain regions in future studies.

## 2. Materials and methods

### 2.1. Animals

A total of 30 male SHRs (age: 14 weeks, weight: 280 ± 20 g) and 10 male Wistar–Kyoto (WKY) rats (age: 14 weeks, weight: 280 ± 20 g) were purchased from Beijing Vital River Laboratory Animal Technology Co., Ltd., (SCXK [Beijing] 2016-0006). All animals were reared at the laboratory of the Beijing University of Chinese Medicine (SPF-grade), and the rearing conditions were uniformly maintained at the laboratory. All rats were adaptively fed for 1 week before the experiment so as to reduce the influence of the rearing environment on the experimental outcomes. All experiments were approved by the Institutional Animal Care and Use Committee of the Beijing University of Chinese Medicine (ethics approval number: BUCM-4-2021041001-2093).

### 2.2. Experimental procedures

**FIGURE 1:**
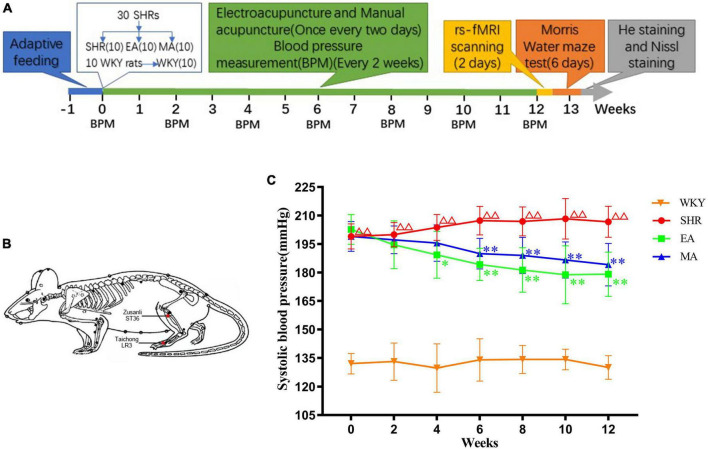
Electroacupuncture and manual acupuncture attenuate hypertension in spontaneously hypertensive rats (SHRs). **(A)** Experimental procedures. **(B)** The location of acupoints in SHRs. Red dots indicate the location of Zusanli (ST36) and Taichong (LR3). **(C)** Systolic blood pressure values in each group. The values are presented as the mean ± SEM (*n* = 10 rats/group). ^ΔΔ^*p* < 0.01 vs. Wistar–Kyoto (WKY) group; *p < 0.05 and ^**^p < 0.01 vs. SHR group. WKY, normal-control group; SHR, model group; EA, electroacupuncture-stimulation group; MA, manual acupuncture-stimulation group.

The experimental process is depicted in Figure 1A. Briefly, after 1 week of adaptive feeding to all rats, 30 SHRs were randomly assigned to the model (SHR), EA, and MA groups, with 10 rats/group. Ten WKY rats served as the blank control (WKY). The rats were intervened every alternate day for 12 weeks. The blood pressure of all rats was recorded every 2 weeks during the intervention period. After the intervention, eight rats from each group were randomly subjected to rs-fMRI scanning. Next, the MWM test was performed to all rats. Finally, the rats were sacrificed for obtaining their brain tissues, and their brain slices were stained with hematoxylin–eosin (HE) and Nissl.

### 2.3. Blood pressure measurement

The blood pressure was measured and recorded at a fixed time (08:00 to 11:00) on days 0, 14, 28, 42, 56, 70, and 84 of the intervention courses. At room temperature (23 ± 2°C), the rats (non-anesthetized) were placed in the blood pressure-measurement rat sleeve (thermostat used to maintain the temperature at 36°C) by two experienced technicians and for a 10-min preheat. The blood pressure was measured in the tail artery of the rats using an indirect blood pressure meter (BP-2010E, Softron Biotechnology, Beijing, China). The systolic blood pressure of each rat was measured thrice and the average value was used as the measurement outcome.

### 2.4. Interventional methods

Interventions were initiated after measurement and the baseline blood pressure of the rats was recorded. The rats in the EA and MA groups (non-anesthetized) were fixed on a sterilized rat plate. For the rats in the two groups, the same acupoints were selected (Figure 1B): Taichong (LR3), which is located at the anterior aspect of the depression between the first and second metatarsal bones, and Zusanli (ST36), which is located at 2-mm lateral to the anterior tubercle of the tibia and 5-mm below the capitulum fibulae under the knee joint (Ma et al., 2020). The sterilized disposable stainless-steel needles (0.18 × 13 mm, ZHONGYANTAIHE, Beijing, China) were inserted into the bilateral LR3 of the rat 2-mm deep at a slope of 30 degrees, and the perpendicular needling was applied at ST36 up to a 5-mm depth (Tian et al., 2013). In the EA group the bilateral acupoints were connected with an acupoint nerve stimulator (Gensun Medical Technology, Jiangsu, China) for current stimulation with an intensity of 1 mA, 2 Hz (Zhang et al., 2009). In the MA group, needles on the bilateral acupoints were bidirectionally rotated for manual stimulation. Each needle was rotated at the speed of 180°/s within 90°. The manipulation was applied every 5 min for 15 s each time (Ding et al., 2019). The EA and MA groups were treated for 20 min each time, the SHR and WKY groups were not treated but were subjected to the same grasping and fixation stimulation similar to that for the EA and MA groups for 20 min each. The interventions were performed every alternate day from 13:00 to 16:00 over 12 weeks.

### 2.5. The MWM test

A day before the formal experiment, the rats were placed in a pool (no platform) for adaptive swimming (60 s) to familiarize themselves with the environment. The MWM experiment was conducted for six consecutive days, from 8:00 to 12:00. We then divided the cylindrical pool (160-cm diameter, 50-cm deep) into four quadrants. In the acquired training group, a circular platform (10-cm diameter) was placed 1–2 cm below the horizontal plane of the target quadrant, and the time taken for the rats to enter the water from different quadrants to find the platform (escape latency) was recorded. The time limit was set to 60 s. A timeout was recorded as 60 s, after which the rats were placed on the platform for 10 s to familiarize themselves with the surrounding environment. Each rat was trained four times a day at an interval of 30 min for five consecutive days. On the 6th day, a space probe trial was performed, and the platform was removed from the pool. We then recorded and observed the time spent by the rats in the target quadrant within the 60-s period, the number of platform crossings, and the swimming traces.

### 2.6. MRI acquisition and data processing

#### 2.6.1. Anesthesia and fixation of rats

Eight rats from each group were randomly subjected to rs-fMRI scanning. Briefly, the rats were injected dexmedetomidine hydrochloride (100 μg/mL) into the interior lateral thigh muscle to prepare them before scanning using a dose of 0.02 mL per 100 g⋅bw. The rats were then anesthetized with a 5% isoflurane and 95% oxygen mixture for induction. During the scanning process, the rats were kept prone on the rat-specific scanning bed, fixed to the head through the hook and ear rods, with a 2% isoflurane and 98% oxygen mixture used for maintaining the anesthetic state. The T2-weighted sequence was scanned first, followed by the BOLD sequence. The body temperature, respiratory rate, and heart rate of the rats were monitored in real-time by a physiological detector (Model 1025, Small Animal Instruments Inc., Stony Brook, NY, USA) during the scanning process.

#### 2.6.2. Scan sequence and parameter setting

All rats were scanned with the 7.0T Bruker animal *in vivo* MRI scanner (PharmaScan 70/16 US, Bruker, Germany) with a special cranial surface coil for small animals. The T2-weighted images were acquired by using the T2_TurboRARE sequence with the following parameters: repetition time (TR) = 5,500 ms, echo time (TE) = 33 ms, percent phase field view = 100, slice thickness = 0.5 mm, acquisition matrix = 256 × 256, flip angle = 90°. The images of resting-state blood oxygen level-dependent (BOLD) were acquired with the T2Star_FID_EPI sequence using the following parameters: TR = 2,000 ms, TE = 11 ms, number of averages = 1, percent phase field view = 80, slice thickness = 0.5 mm, acquisition matrix = 80 × 48, and flip angle = 90°.

#### 2.6.3. Data preprocessing and parameters calculation

SPM12 package, REST package, FC toolkit, and DPABI package on the MATLAB platform were used for data preprocessing and the calculation of indicators. (1) Format conversion: The original functional image and T2 image were converted from the DICOM format to the NIFTI format. (2) The removal of the starting time point: the BOLD signal is often unstable at the beginning of functional image acquisition; therefore, the first 10 time points have to be excluded. (3) Voxel augment: The SPM package is designed based on the actual size of the human brain. The mouse brain is much smaller than the human brain; therefore, the collected rat MRI was enlarged 10 times to adapt to the operation of the package. (4) Slice timing: The data of all voxels were adjusted to the standard time so as to achieve theoretical consistency of the acquisition time of BOLD signals for all voxels at a time point. (5) Realign: The little movement of each rat’s head was aligned between different time points in the scan. The criterion for exclusion was set as one voxel size movement (3 × 3 × 3 mm after amplification). (6) Reorientation: the average functional phase was corrected for origin, and the origin correction matrix of the average functional phase was applied to the functional phase file. The T2 phase was also corrected for the origin. (7) Normalization: all subject brains were registered to a unified standard space to resolve the problems of the difference in the brain shape between different rats and the inconsistency in the head spatial position during scanning so as to facilitate subsequent statistical analyses. (8) Smooth: the high-frequency noise generated by image deformation during spatial standardization was reduced and the statistical effectiveness was improved. The size of the smoothing nucleus was 2–3 times the size of the enlarged voxel. (9) Indicator calculation: The REST package and the SPM12 package were used in the calculation of the ALFF and ReHo values. The ALFF data calculation was performed in the 0.01–0.08-Hz frequency band after the spatial-smoothing process. The ReHo data calculation was performed as per the spatial normalization process, and the noise in the frequency band < 0.01 Hz (low frequency) and > 0.08 Hz (high frequency) was removed. The FC package, the SPM12 package, and the DPABI package were employed in the calculation of the FC values performed after the spatial smoothing process.

### 2.7. Histopathology

Six rats were randomly selected from each group. From them, samples of three rats were subjected to HE staining, and the other three to Nissl stanning. These rats received deep anesthesia and precooled normal saline and 4% paraformaldehyde were perfused through the cardiac veins. The brain tissues were collected and fixed in a 4% paraformaldehyde solution. The tissue was dehydrated, subjected to transparent treatment in xylene, embedded in paraffin wax, sliced into 4 μm slices, flattened, and stored after baking.

HE staining: The sections were dewaxed, covered with water, stained with hematoxylin for 3–8 min, and differentiated in the hydrochloric acid alcohol solution. Then, the slices were stained with eosin for 1–3 min, dehydrated in graded ethanol, treated with xylene, and sealed with neutral resin. The sections were observed with a panoramic scanner (Pannoramic MIDI, 3D HISTECH, Budapest, Hungary) and the relevant images were collected.

Nissl staining: The sections were dewaxed and covered with water, stained with Nissl stain for 10–30 min, differentiated in the Nissl differentiation fluid for 1–3 min, dehydrated in graded ethanol, treated with xylene, and sealed with neutral resin. The sections were observed in a panoramic scanner (Pannoramic MIDI, 3D HISTECH, Budapest, Hungary) and the relevant images were collected. We then randomly selected five fields (Scale bar = 100 μm) in the entorhinal cortex of each brain section. The number of nissl-positive neurons was calculated with the ImageJ software (version 1.8, National Institute of Health, Bethesda, MD, USA), and the average number of the cells in the five fields was counted.

### 2.8. Statistical analyses

The experimental data were analyzed by the SPSS 20.0 software (IBM, Armonk, NY, USA). The normality of the data was tested, followed by the homogeneity test. The experimental data on the systolic blood pressure and escape latency in the MWM test was subjected to a two-way repeated measurement analysis of variance. The experimental data on the time spent in the target quadrant, the number of platform crossings, and the number of Nissl-positive neurons were analyzed by one-way analysis of variance. Fisher’s least-significant difference (LSD) test was performed for pairwise comparison between the groups, and *p* < 0.05 was considered to indicate statistical significance. Line charts and histograms in the experiment were drawn with GraphPad Prism 8 (GraphPad Software, San Diego, CA, USA).

The rs-fMRI data was modeled in the general linear model. The data were compared between groups by one-way analysis of variance, followed by a *post hoc* two-sample *t*-test. The regions with significant changes in the ALFF and ReHo values were considered as uncorrected *p* < 0.005 with cluster-extent > 5 voxels. The regions with significant changes in the FC values were considered as uncorrected *p* < 0.005 with cluster-extent > 2 voxels.

We performed Pearson’s (data conform to the normal distribution) or Spearman’s (data did not conform to the normal distribution) correlation analyses between the behavioral test indices (the escape latency and the time spent in the target quadrant) and ALLF/ReHo values in the regions with significant changes in the EA/MA group. We consider *p* < 0.05 as a threshold of statistical significance.

## 3. Results

### 3.1. Effects of EA and MA on blood pressure in SHRs

We analyzed systolic blood pressure values of SHRs to investigate the attenuating effects of EA and MA on hypertension. The results demonstrated significant treatment effect (*F* = 582.178, *p* < 0.001), time effect (*F* = 3.548, *p* < 0.01), and interaction of time and treatment effect (*F* = 4.301, *p* < 0.001), indicating significant differences in the systolic blood pressure values of each group in different periods. When compared with the WKY group, the systolic blood pressure of the SHR group gradually increased and was stable at a higher level within 0–12 weeks of intervention (*p* < 0.01). Compared with the SHR group, the systolic blood pressure of the EA and MA groups began to decrease at the 4th and 6th week of measurement, respectively, (*p* < 0.05, *p* < 0.01). Specifically, when compared with the SHR group, the systolic blood pressure of both the EA and MA groups decreased consecutively from the 6th week to the 12th week of intervention (both *p* < 0.01). Thus, both EA and MA can reduce systolic blood pressure in SHRs, and the acupuncture effect in lowering blood pressure has a cumulative time effect. Moreover, EA exhibited a rapid onset in lowering the blood pressure when compared with MA (Figure 1C).

### 3.2. Effects of EA and MA on cognitive functions of SHRs

**FIGURE 2:**
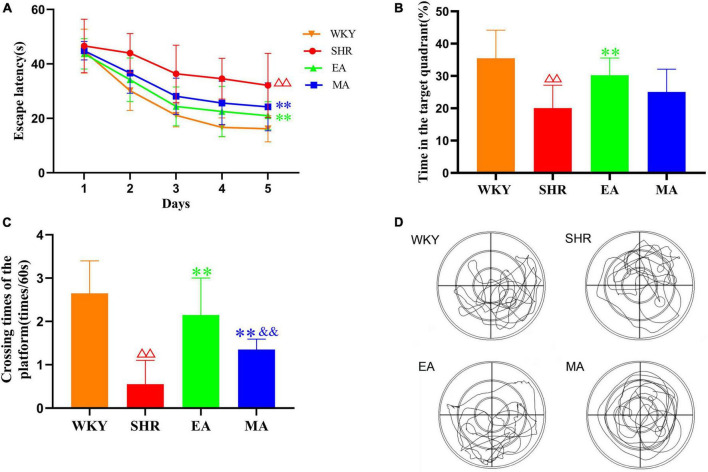
Electroacupuncture and manual acupuncture protect the cognitive functions of spontaneously hypertensive rats (SHRs). The Morris water maze test was performed in the Wistar–Kyoto (WKY), SHR, EA, and MA groups. **(A)** Escape latency. **(B)** Time spent in the target quadrant. **(C)** The number of platform crossings. The values are presented as the mean ± SEM (*n* = 10 rats/group). **(D)** Typical swimming traces in the probe trial. ^ΔΔ^*p* < 0.01 vs. WKY group; ^**^p < 0.01 vs. SHR group; ^&&^*p* < 0.01 vs. EA group. WKY, normal control group; SHR, model group; EA, electroacupuncture group; MA, manual acupuncture group.

Morris water maze (MWM) reflected the spatial learning and memory function of rats. In the acquired training experiment (Figure 2A), the escape latency had a significant treatment effect (*F* = 24.113, *p* < 0.001) and time effect (*F* = 59.745, *p* < 0.001). The SHR group required more time to find the hidden platform than the WKY group (*p* < 0.01). On the contrary, the EA and MA groups required a significantly shorter time (both *p* < 0.01) to identify the platform when compared to the SHR group. In the probe trial (Figures 2B–D), time spent in the target quadrant and the number of platform crossings were significantly reduced in the SHR group when compared with the WKY group (both *p* < 0.01). Time spent in the target quadrant in the EA group was significantly increased when compared with that in the SHR group (*p* < 0.01). No significant difference was observed between the MA and SHR groups (*p* > 0.05). When compared with the SHR group, the number of platform crossings was significantly increased in the EA and MA groups (both *p* < 0.01). Specifically, the number of platform crossings was higher in the EA group than in the MA group (*p* < 0.01). Thus, chronic hypertension can cause impairment of learning and memory function. EA and MA exhibited different degrees of attenuating effects on cognitive impairment followed by hypertension, and comprehensive analysis revealed that the effect of EA was better than that of MA.

### 3.3. Effects of EA and MA on brain regions regulating blood pressure and cognitive functions of SHRs

**FIGURE 3:**
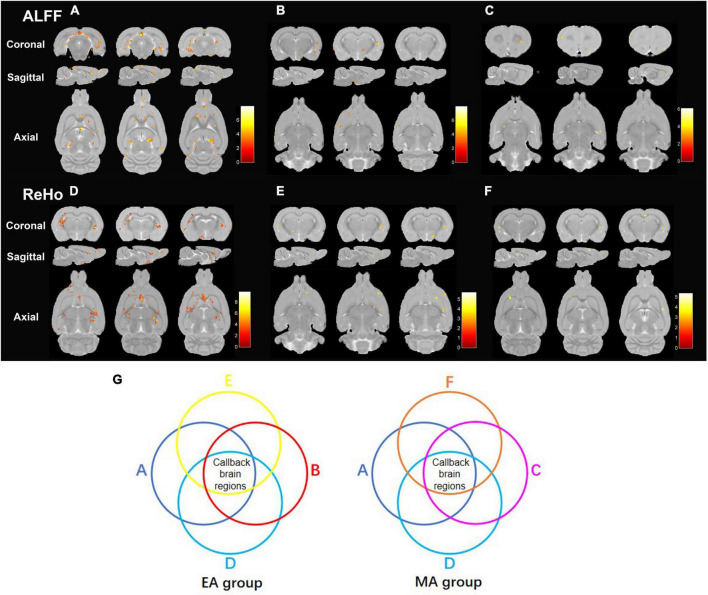
Electroacupuncture and manual acupuncture activate callback brain regions regulating blood pressure and cognitive functions of spontaneously hypertensive rats (SHRs). ALFF and ReHo analyses were performed in the Wistar–Kyoto (WKY), SHR, EA, and MA groups. **(A)** The brain regions with decreased ALFF values in SHR compared to that in WKY. **(B)** The brain regions with increased ALFF values in EA compared to that in SHR. **(C)** The brain regions with increased ALFF values in MA compared to that in SHR. **(D)** The brain regions with decreased ReHo values in SHR compared to that in WKY. **(E)** The brain regions with increased ReHo values in EA compared to that in SHR. **(F)** The brain regions with increased ReHo values in MA compared to that in SHR. Differential brain regions are displayed sequentially on the coronal plane, sagittal plane, and axial plane (*p* < 0.005, uncorrected, Cluster > 5). Color bars signify the *t* value of the group analysis (brighter color represents a higher *t* value). **(G)** The process of determining the callback brain regions. WKY, normal-control group; SHR, model group; EA, electroacupuncture group; MA, manual acupuncture group; ALFF, the amplitude of low-frequency fluctuation; ReHo, regional homogeneity.

Data accessed through rs-fMRI were subjected to ALFF and ReHo analyses. ALFF (Supplementary Table 1) and ReHo (Supplementary Table 2) values of some brain regions were significantly lower in the SHR group than in the WKY group (Figures 3A, D), indicating that chronic hypertension decreased the functional activity in some brain areas. ALFF (Figures 3B, C; Supplementary Tables 3, 4) and ReHo (Figures 3E, F; Supplementary Tables 5, 6) values of some brain regions were significantly higher in the EA and MA groups than in the SHR group, demonstrating that EA and MA activated these brain areas and enhanced the functional activity of local neurons. After ALFF and ReHo analyses, we performed an intersection between the brain regions of decreased ALFF/ReHo values in the SHR group (compared to the WKY group) and the brain regions of increased ALFF/ReHo values in the EA group (compared to the SHR group) to obtain reversal brain regions of the EA group, that is, the callback brain regions of the EA group (Figure 3G). These regions included the right hypothalamus region, right brainstem, right entorhinal cortex, right prelimbic cortex, right basal forebrain region, bilateral cerebellum, right olfactory bulb, and right primary somatosensory cortex. In the same manner, we obtained the callback brain regions of the MA group. These regions included the right hypothalamus region, right entorhinal cortex, right cingulate cortex, right corpus callosum, right striatum, and bilateral olfactory bulb. The EA group activated more callback brain regions than the MA group, and most of these regions were involved in regulating blood pressure and cognitive functions. In addition, the brain areas regulating the sensation and olfaction were activated. These areas are often considered the key targets for acupuncture intervention.

### 3.4. Effects of EA and MA on functional connectivity between seeds and brain regions regulating blood pressure and cognitive functions of SHRs

Based on the results of ALFF and ReHo analyses, we performed another intersection between the callback regions of the EA group and the MA group, in which the right hypothalamus (HHA. R) and the right entorhinal cortex (Ent. R) were identified, indicating a critical role of the functional activity of the two brain regions. Therefore, the right hypothalamus (HHA. R) and right entorhinal cortex (Ent. R) were selected as seeds for FC analysis. When compared with the WKY group, the functional connectivity of HHA.R (Supplementary Table 7) and Ent. R (Supplementary Table 8) in the SHR group with other brain regions was impaired, indicating that chronic hypertension damaged functional activity in brain regions as well as the functional connectivity between them. EA and MA exerted reversal effects as they enhanced functional connectivity between the seeds and some connected brain regions (Table 1). Most brain areas were involved in blood pressure and cognitive function regulation. In addition, the reversal of functional connectivity in the EA group partially coincided with that in the MA group, and the number of callback brain regions functionally connected with seed points was higher in the EA group than in the MA group.

**TABLE 1:** The increased strengths of the functional connectivity (FC) in the electroacupuncture (EA), and the manual acupuncture (MA) groups in comparison with the spontaneously hypertensive rats (SHR) group.

Connected region	Group	Positive brain regions	Voxel size	*t* value	Peak MNI coordinate (mm)		
					***X***	***Y***	***Z***
Seed 1 HHA.R	EA group	Brainstem. R	2	3.53	8.00	-101.05	-14.80
		Entorhinal cortex.L	2	3.70	-52.00	-35.05	-11.80
		Molecular layer of the cerebellum.R	3	4.44	41.00	-125.05	18.20
	MA group	Brainstem. L	2	3.72	-13.00	-92.05	-32.80
		Molecular layer of the cerebellum.R	2	4.79	32.00	-125.05	27.20
		Basal forebrain region. R	2	4.41	14.00	24.95	-11.80
Seed 2 Ent.R	EA group	Cornu ammonis 1.R	2	4.10	23.00	-47.05	51.20
		PreLimbic cortex.R	2	4.38	16.00	33.95	15.20
		Dentate gyrus.R	4	3.24	32.00	-59.05	36.20
		Brainstem. R	2	3.97	20.00	-125.05	-26.80
		Corpus callosum. R	2	4.10	23.00	-47.05	51.20
		Striatum.R	3	3.14	23.00	12.95	21.20
		Granule cell level of the cerebellum.L	2	4.96	-64.00	-113.05	6.20
	MA group	Cornu ammonis 1.R	2	4.26	50.00	-56.05	6.20
		Dentate gyrus.R	2	4.26	50.00	-56.05	6.20
		Ectorhinal cortex.L	2	4.14	-58.00	-32.05	0.20
		Brainstem. L	3	3.83	-1.00	-50.05	-5.80
		Granule cell level of the cerebellum.L	2	3.34	-28.00	-122.05	12.20

HHA.R, hypothalamic region.R; Ent.R, entorhinal cortex.R; FC, functional connectivity; EA, electroacupuncture group; MA, manual acupuncture group; L, left; R, right. *p* < 0.005, uncorrected, Cluster > 2.

### 3.5. Relationship between the ALLF/ReHo value of right entorhinal cortex and behavioral test indices in MWM test

**FIGURE 4:**
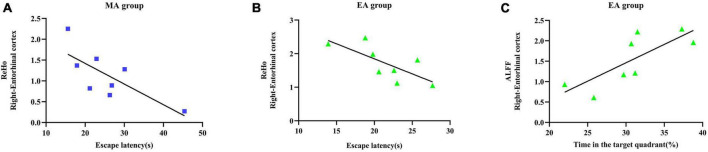
Relationship between functional activity of entorhinal cortex and behavioral test indices of Morris water maze (MWM) test. **(A)** Correlation analysis between the regional homogeneity (ReHo) value of entorhinal cortex and escape latency in the manual acupuncture (MA) group. **(B)** Correlation analysis between the ReHo value of entorhinal cortex and escape latency in the electroacupuncture (EA) group. **(C)** Correlation analysis between the amplitude of low-frequency fluctuation (ALFF) value and the time spent in the target quadrant in the EA group. *n* = 8 per group. Pearson’s correlation analysis was used with *p* < 0.05 for statistical significance and r for correlation.

The results of Pearson’s correlation analysis (Figures 4A–C) revealed significant correlations between the ReHo value of Ent. R in the MA group and the escape latency (*r* = −0.752, *p* = 0.031), the ReHo value of Ent. R in the EA group and the escape latency (*r* = 0.738, *p* = 0.037), and the ALFF value of Ent. R in the EA group and the time spent in the target quadrant (*r* = 0.776, *p* = 0.024). Specifically, the ReHo value of Ent. R in the MA/EA group was negatively correlated with the escape latency; the ALFF value of Ent. R in the EA group was positively correlated with the time spent in the target quadrant. These results demonstrated that under the intervention of the EA and the MA, the stronger the functional activity of entorhinal cortex, the better was the performance of MWM test of the rats.

### 3.6. Effects of EA and MA on neuronal injury in entorhinal cortex in SHRs

**FIGURE 5:**
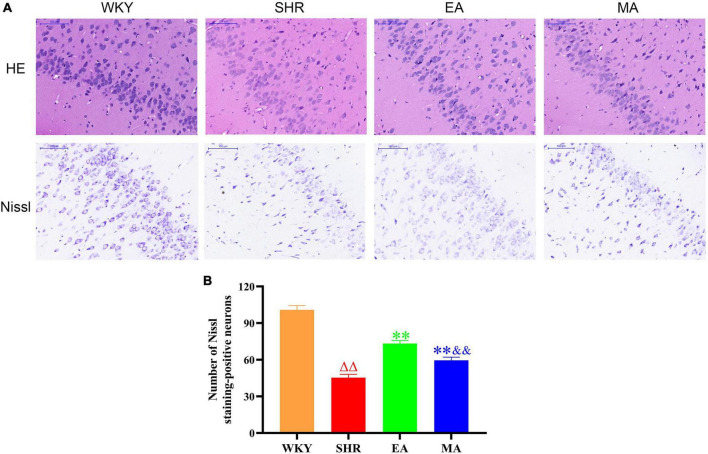
Electroacupuncture and manual acupuncture ameliorate neuronal injury in entorhinal cortex in spontaneously hypertensive rats (SHRs). The results of HE staining and Nissl staining in the entorhinal cortex. **(A)** Representative images of HE staining and Nissl staining in the entorhinal cortex of the Wistar–Kyoto (WKY), SHR, EA, and MA groups. Scale bar = 100 μm. **(B)** Quantitative evaluation of Nissl staining-positive neurons. Scale bar = 100 μm. The values are expressed as the means ± SEM (*n* = 3 rats/group). ^ΔΔ^*p* < 0.01 vs. WKY group; ^**^p < 0.01 vs. SHR group; ^&&^*p* < 0.01 vs. EA group. WKY, normal control group; SHR, model group; EA, electroacupuncture group; MA, manual acupuncture group.

Through HE staining, histopathological changes in the entorhinal cortex were evaluated. Neurons in the WKY group were histologically normal; they were closely arranged and orderly and exhibited a regular morphology, with uniform size. In contrast, neurons in the SHR group were disordered, with increased intercellular space. Furthermore, neuronal injury and apoptosis occurred. However, when compared with the SHR group, the EA and MA groups improved these abnormalities. The EA group specifically exhibited a better improvement than the MA group (Figure 5A, 1st line). The number of Nissl-positive neurons in the entorhinal cortex was also evaluated using Nissl staining. The number of Nissl-positive neurons was significantly decreased in the SHR group when compared with that in the WKY group (*p* < 0.01). The number of Nissl-positive neurons was significantly increased in the EA and MA groups compared with the SHR group (*p* < 0.01). Moreover, the EA group had more Nissl-positive neurons than the MA group (*p* < 0.01) (Figures 5A, B).

## 4. Discussion

In this study, 14-weeks-old SHRs were selected as the experimental model. SHRs are internationally recognized animal models of hypertension (Tayebati et al., 2012). They are often used in studies on hypertensive brain injury as their symptoms are similar to those of humans, such as course-dependent arterial high blood pressure, brain atrophy, and brain neuron loss (Amenta et al., 2010; Fei et al., 2017; Gao et al., 2019). SHRs exhibit stable hypertensive symptoms at approximately 12 weeks of age and cognitive impairment at 26–28 weeks of age (Johnson et al., 2020). Clinical studies (Turana et al., 2019; de Menezes et al., 2021) have confirmed that ameliorating hypertension in the early stage is crucial for protecting cognitive functions. SHRs aged 14 weeks are in the early hypertension stage and exhibit no cognitive impairment. Therefore, 14-weeks-aged SHRs are suitable for investigating the attenuating effects of acupuncture in cognitive dysfunction. According to the traditional Chinese medicine theory, several acupoints control blood pressure. Of them, LR3 and ST36 are two commonly used acupoints that lower the blood pressure in clinical and animal studies (Lai et al., 2012; Zhang et al., 2018; Luo et al., 2019). Leung et al. (2016) reported that LR3 and ST36, when combined, are beneficial for protecting cognitive functions in SHRs.

In this study, the blood pressure of SHRs exhibited a slowly increasing trend within the experimental period, which is similar to hypertension development in humans (Philip et al., 2021). Both EA and MA could ameliorate blood pressure in SHRs, demonstrating consistency with the results of the previous reports of our team (Wu et al., 2021; Yang et al., 2022a). Moreover, EA has a faster onset of the blood pressure-lowering effect than MA. Interestingly, we found that blood pressure amelioration presented a cumulative effect with progressing EA and MA interventions. In fact, the long-term cardiovascular effect of an acupuncture therapy course is considerably better than that of one-time acupuncture (Longhurst and Tjen, 2013). This agrees with the result of Li et al. (2018). They reported the cumulative effect of acupuncture at LR3 on lowering blood pressure. Clinical and animal studies (Lu et al., 2017; Whelton and Williams, 2018) have proven that antihypertensive treatment protects against cognitive impairment. The MWM test results indicated the attenuating effect of EA and MA on cognitive impairment in hypertensive rats, which may be related to the antihypertensive effect of acupuncture.

A past study has confirmed that acupuncture can exert therapeutic effect through a series of pathways of action (Lai et al., 2012). ALFF and ReHo analyses revealed that the callback brain regions of EA and MA are mainly involved in regulating blood pressure and cognitive functions. The callback brain regions shared by the two interventions are the hypothalamus and the entorhinal cortex. The functional activities of these two brain regions have crucial roles in lowering blood pressure and protecting cognitive functions. The hypothalamus is a key brain region associated with blood pressure regulation. Combined with the brainstem, the hypothalamus regulates sympathetic nerve activity (Farnham et al., 2008; Ye et al., 2013), which is often related to essential hypertension occurrence (Guyenet, 2006). Moreover, angiotensin secreted by astrocytes that is present in both the hypothalamus and brainstem (Bloch et al., 2015) plays a major role in blood pressure regulation. In addition, the hypothalamus regulates cardiovascular and cerebrovascular effects through oxidative stress pathways (Coleman et al., 2013), proinflammatory cytokine levels (Qi et al., 2013), and arginine vasopressin secretion (Yi et al., 2012). The entorhinal cortex is a key player in brain learning and memory during memory storage, consolidation, and reactivation (O’Neill et al., 2017; Gerlei et al., 2021). The entorhinal cortex selectively gatekeeps the cortical memory network by interacting with brain regions, such as the anterior cingulate cortex or hippocampus, selectively according to the age of memory (Takehara-Nishiuchi, 2014). Hales et al. (2021) found that the performance of rats with entorhinal cortex lesions is unsatisfactory in remembering platform positions in the MWM test. Imaging studies (Zuo et al., 2018; Wang et al., 2020) have revealed that the functional connectivity between the entorhinal cortex and basal forebrain regions is impaired in cognitive dysfunction patients. In addition, other callback brain regions such as the prelimbic cortex (da Silva et al., 2020), cingulate cortex (Rolls, 2019), corpus callosum (Edwards et al., 2014), cerebellum (Argyropoulos et al., 2020), and striatum (Provost et al., 2015) are associated with advance cognitive activities such as learning, memory, and emotion. Some fMRI studies (Wen et al., 2018; Wei et al., 2020) have reported that EA and MA exert a therapeutic role by activating the functional activities of the brain regions such as the prelimbic cortex, cingulate cortex, and cerebellum. A past study also substantiated the regulatory effect of acupuncture on the functional activities of the cerebral cortex and some subcortical regions (Cai et al., 2018). In this study, acupuncture activated the neuronal functional activity of the brain regions that regulate blood pressure and cognitive function, indicating that the activation may act as one of the neuroimaging mechanisms underlying the ameliorating effect of acupuncture on hypertension and cognitive impairment. In addition, the brain regions regulating sensation and olfaction were activated, which may have positive effects on attenuating the occurrence and development of hypertensive brain injury.

Based on the results of the previously mentioned analysis, the hypothalamus and entorhinal cortex were selected as seeds in the FC analysis. According to the results, acupuncture enhanced the functional connectivity between the seeds and brain regions such as the brainstem, entorhinal cortex, hippocampus, prelimbic cortex, and cerebellum. Chen et al. (2013) reported that acupuncture has a regulatory role in the cardiovascular system as it affects complex brain networks such as the cerebral cortex, hypothalamus, and brainstem, which is similar to the results of our analysis. Interestingly, EA enhanced the functional connectivity between the hypothalamus, the selected seed point, and the entorhinal cortex, thereby suggesting a close relationship between blood pressure and cognitive functions. In addition, when the entorhinal cortex was used as the seed point, functional connectivity between the seed points (both in the EA and MA groups) and hippocampus (CA1 area and dentate gyrus) were enhanced. Projection neurons in the entorhinal cortex directly formed excitatory synapses on the pyramidal cells of the CA1 area for impulse transmission (Li et al., 2017). Deep brain stimulation of the entorhinal cortex increases dentate gyrus neurogenesis (Jiang et al., 2022). An MRI study (Lin et al., 2022) proved that EA improves cognitive function by increasing the functional connectivity between the entorhinal cortex and hippocampus in mice. Thus, the neurofunctional connection among the hypothalamus, entorhinal cortex, and hippocampus may play a critical role in regulating blood pressure and cognitive functions.

The hippocampus is crucial for learning and memory (Lisman et al., 2017). When compared with the WKY group, ALFF and ReHo values of the hippocampus in the SHR group decreased, suggesting that chronic hypertension damaged the functional activity of hippocampal neurons. However, the hippocampus is not one of the EA- and MA-activated callback brain areas. In fact, a lot of information from the cortex is projected from the entorhinal cortex to the hippocampus (Jiang et al., 2022). A study reported that entorhinal cortical atrophy in Parkinson’s disease patients with dementia occurs earlier than hippocampal atrophy (Goldman et al., 2012). Therefore, acupuncture may activate the entorhinal cortex earlier than the hippocampus. The FC analysis revealed that the functional connectivity between the entorhinal cortex and hippocampus was enhanced. In this study, there was a significant correlation between the functional activities of the entorhinal cortex in the MA/EA group and the indices of MWM test. It not only explains the curative effect of acupuncture, but also further illustrates the importance of the entorhinal cortex in regulating the cognitive functions. Histopathologically, acupuncture exhibited an attenuating effect on neuronal damage and apoptosis in the entorhinal cortex. This finding illustrates the synergy of acupuncture in protecting neuronal structure and functional activities.

Electroacupuncture (EA) was superior to MA in lowering blood pressure and protecting cognitive functions in SHRs. ALFF and ReHo analyses revealed that the callback brain regions activated by the EA and MA groups were duplicated but not identical, and that the total number of callback brain regions activated was higher in the EA group than in the MA group. Similarly, FC analysis demonstrated that a higher number of callback brain regions in the EA group were involved in functional connectivity enhancement when compared with the MA group. In addition, histopathological analysis revealed that EA had a better attenuating effect on entorhinal cortex neuronal injury and apoptosis than MA. In other words, EA activated more callback brain regions and functional connectivity than MA, which may explain the better effect of the EA group relative to that of the MA group.

This study has several limitations. First, as a neuroimaging study, this experiment mainly focused on macro changes in the brain and explored the effect of acupuncture on the brain region functions. The molecular mechanisms were not discussed. Future studies will adopt proteomics and genomics to study the central mechanism of the target brain regions. Second, anesthesia was required during the rs-fMRI scan in rats. Although isoflurane-dexmedetomidine hydrochloride is deemed suitable as an anesthetic method for rs-fMRI (Paasonen et al., 2018), it may affect the rs-fMRI scanning data. Third, only the systolic blood pressure of SHRs was analyzed. In the future, we plan to analyze the diastolic blood pressure data of SHRs to further contribute to the blood pressure research.

## 5. Conclusion

We here confirmed that EA and MA exerted attenuating effects on hypertension and cognitive dysfunction in SHRs, and that the effect of EA is better than that of MA. Neuroimaging analysis revealed that acupuncture enhances the neuronal activity and functional connectivity in the brain regions involved in the regulation of blood pressure and cognitive functions. Specifically, EA activated more callback brain regions and functional connectivity than MA.

## Data availability statement

The original contributions presented in this study are included in the article/Supplementary material, further inquiries can be directed to the corresponding authors.

## Ethics statement

This animal study was reviewed and approved by Institutional Animal Care and Use Committee of Beijing University of Chinese Medicine.

## Author contributions

J-PL participated in the acupuncture intervention, the aggregation of the results of statistical analysis, and drafted the manuscript. Y-YL and K-ZY have performed the measurement and record of the blood pressure of the rats. X-LL, JS, and YG took part in the behavioral experiment and pathological staining of the rats. B-NY took part in the imaging processing. ZT and X-YG took part of the work in statistical analyses. YT participated in the translation, revision, and proofreading of the manuscript. Q-GL, MX, and S-FS responsible for experimental designing and supervision. All authors have reviewed and approved the manuscript.
