# Supplementary material for: Electroacupuncture and manual acupuncture at LR3 and ST36 have attenuating effects on hypertension and subsequent cognitive dysfunction in spontaneously hypertensive rats: A preliminary resting-state functional magnetic resonance imaging study
Source: Front Neurosci. 2023 Mar 9;17:1129688. doi: 10.3389/fnins.2023.1129688 (PMC10033598; doi:10.3389/fnins.2023.1129688)
Supplement: Supplementary file 1 [file Data_Sheet_1.docx]

Supplementary Material

Electroacupuncture and Manual Acupuncture at LR3 and ST36 Exhibit Attenuating Effects on Hypertension and Followed Cognitive Dysfunction in Spontaneously Hypertensive Rats: A Preliminary Resting State Functional Magnetic Resonance Imaging Study

Ji-peng Liu, Yin-yin Li, Ke-zhen Yang, Shu-feng Shi, Yu Gong, Zhuang Tao, Yi Tong, Jiao Sun, Xiao-li Wu, Bing-nan Yue, Xiao-lu Li, Xin-yu Gao, Qing-guo Liu^*^, Meng Xu^*^

*** Correspondence:** Qing-guo Liu: liuqingguo888@vip.sina.com; Meng Xu: chilli.xu@163.com

**Table S1. Brain regions with decreased ALFF values in SHR group compared to that in WKY group**

| Negative brain regions | Voxel size | *t*-value | Peak MNI coordinate (mm) | | |
| --- | --- | --- | --- | --- | --- |
|  |  |  | **X** | **Y** | **Z** |
| Subiculum_R  Cornu_Ammonis_1_R | 6 | -4.893 | 38 | -50.05 | -17.8 |
| Dentate_Gyrus_L  Cornu_Ammonis_3_L | 5 | -3.4724 | -28 | -44.05 | -20.8 |
| Entorhinal_Cortex_R  Amygdalopiriform_Cortex_R  Lateral_Entorhinal_Cortex_external_part_R | 13 | -4.1628 | 65 | -32.05 | -8.8 |
| Basal_Forebrain_Region_R | 10 | -5.3944 | 8 | 12.95 | -17.8 |
| Posterior_Agralunar_Insular_Cortex_L  Entorhinal_Cortex_L  Dysgranular_Insular_Cortex_L | 63 | -6.2777 | -58 | -26.05 | 3.2 |
| Dentate_Gyrus_R | 11 | -5.6316 | 29 | -62.05 | -11.8 |
| Lateral_Entorhinal_Cortex_R  Lateral_Entorhinal_Cortex_Internal_part_R | 6 | -6.3978 | 74 | -56.05 | -8.8 |
| Subiculum_L  Perirhinal_Area__L  Cornu_Ammonis_1_L | 53 | -5.4235 | -58 | -62.05 | 3.2 |
| Corpus_Callosum_and_Associated_Subcortical_White_Matter_R  Perirhinal_Area__R | 78 | -6.1941 | 62 | -74.05 | 15.2 |
| Basal_Forebrain_Regio_L | 11 | -5.0742 | -1 | 33.95 | -2.8 |
| Periventricular_Grey_R  Brainstem_R | 6 | -3.8662 | 5 | -116.05 | 3.2 |
| Parasubiculum_R  Perirhinal_Area_R | 12 | -4.6136 | 38 | -65.05 | 3.2 |
| Secondary_Auditory_Cortex_Ventral_Part_R  Primary_Auditory_Cortex_R | 20 | -5.8004 | 68 | -38.05 | 12.2 |
| Secondary_Auditory_Cortex_Dorsal_Part_R  Posterior_Agralunar_Insular_Cortex_R | 6 | -4.73 | 62 | -26.05 | 3.2 |
| Lateral_Primary_Auditory_Cortex_R  Dysgranular_Insular_Cortex_R | 6 | -4.0709 | 65 | -11.05 | 3.2 |
| Descending_Corticofugal_Pathways_and_Globus_Pallidum_R  Fimbria_of_the_Hippocampus3_R | 11 | -4.5362 | 44 | -20.05 | 6.2 |
| Granule_Cell_Level_of_the_Cerebellum_L | 29 | -5.4691 | -43 | -92.05 | 9.2 |
| Brainstem_L | 5 | -3.39 | -19 | -44.05 | 9.2 |
| Corpus_Callosum_and_Associated_Subcortical_White_Matter_L Descending_Corticofugal_Pathways_and_Globus_Pallidum_L  Primary_Auditory_Cortex_L | 54 | -5.422 | -49 | -23.05 | 18.2 |
| Lateral_Primary_Auditory_Cortex_L  Secondary_Somatosensory_Cortex_L | 11 | -4.6292 | -61 | -5.04999 | 9.2 |
| Primary_Cingular_Cortex_R  PreLimbic_Cortex_R  Septal_Region_L | 84 | -7.0894 | 5 | 27.95 | 24.2 |
| Olfactory_Bulb_R | 9 | -4.0207 | 8 | 51.95 | 12.2 |
| Hypothalamic_Region_R  Anterior_Commissure_Intrabulbar_part_R | 12 | -5.0571 | 11 | 66.95 | 9.2 |
| Parasubiculum_L  Perirhinal_Area_35_L | 51 | -5.4631 | -43 | -53.05 | 33.2 |
| Granule_Cell_Level_of_the_Cerebellum_L | 5 | -3.7838 | 20-16 | -122.05 | 15.2 |
| Molecular_Layer_of_the_Cerebellum_L | 5 | -4.6005 | -58 | -125.05 | 18.2 |
| Molecular_Layer_of_the_Cerebellum_R  Granule_Cell_Level_of_the_Cerebellum_R | 16 | -5.7014 | 20 | -95.05 | 21.2 |
| Primary_Somatosensory_Cortex_Upperlips_R  Primary_Somatosensory_Cortex_Barrel_field_R | 33 | -4.972 | 50 | -20.05 | 24.2 |
| Agranular_Insular_Cortex_R | 17 | -5.9332 | 35 | 24.95 | 21.2 |
| Perirhinal_Area_36_L | 7 | -3.5726 | -46 | -80.05 | 24.2 |
| Thalamus__L | 5 | -3.5593 | -34 | -35.05 | 21.2 |
| Cornu_Ammonis_3_R | 11 | -3.699 | 41 | -59.05 | 27.2 |
| Ectorhinal_Cortex_R | 6 | -3.9494 | 68 | -80.05 | 27.2 |
| Superficial_Gray_Layer_of_the_Superior_Colliculus_L  Deeper_Layers_of_the_Superior_Colliculus_L | 16 | -3.8346 | -25 | -56.05 | 24.2 |
| Ventral_Hippocampal_Commissure_R  Ventral_Hippocampal_Commissure_L | 15 | -5.7001 | 2 | -11.05 | 27.2 |
| Striatum_R | 5 | -3.8516 | 29 | 6.95001 | 24.2 |
| Pretectal_Region_L | 6 | -3.608 | -1 | -44.05 | 27.2 |
| Thalamus__R  Fimbria_of_the_Hippocampus2_R | 5 | -3.7742 | 35 | -29.05 | 27.2 |
| Primary_Motor_Cortex_L  Orbitofrontal_Region_L  Secondary_Cingular_Cortex_L | 36 | -6.0273 | -19 | 27.95 | 36.2 |
| Superficial_Gray_Layer_of_the_Superior_Colliculus_R  Deeper_Layers_of_the_Superior_Colliculus_R | 46 | -6.0645 | 26 | -44.05 | 33.2 |
| Glomerular_Layer_of_the_Olfactory_Bulb_R | 18 | -5.5126 | 5 | 81.95 | 36.2 |
| Perirhinal_Cortex_L  Retosplenial_Dysgranular_Cortex_L | 7 | -4.1667 | -46 | -95.05 | 36.2 |
| Retrosplenial_Granular_Cortex_Part_A_L  Retrosplenial_Granular_Cortex_Part_A_R  External_Cortex_of_the_Inferior_Colliculus__R | 239 | -7.9096 | 14 | -80.05 | 57.2 |
| Superficial_Gray_Layer_of_the_Superior_Colliculus_L | 6 | -5.5498 | -22 | -50.05 | 36.2 |
| Primary_Somatosensory_Cortex_Forelimb_R  Primary_Somatosensory_Cortex_Barrel_field_R | 28 | -5.0772 | 35 | -2.04999 | 42.2 |
| Corpus_Callosum_and_Associated_Subcortical_White_Matter_L  Striatum_L | 5 | -4.6233 | -22 | 15.95 | 33.2 |
| Primary_Somatosensory_Cortex_Jaw_R  Orbitofrontal_Region_R | 6 | -4.6077 | 32 | 33.95 | 33.2 |
| Frontal_Association_Cortex_L  Frontal_Association_Cortex_R | 23 | -3.9182 | 5 | 57.95 | 36.2 |
| Molecular_Layer_of_the_Cerebellum_R  Perirhinal_Cortex_R | 5 | -3.8137 | 47 | -98.05 | 39.2 |
| Secondary_Cingular_Cortex_R | 6 | -4.0323 | 11 | 9.95001 | 39.2 |
| Primary_Motor_Cortex_L | 12 | -4.7501 | -25 | 15.95 | 45.2 |
| Molecular_Layer_of_the_Cerebellum_L | 26 | -4.8878 | -40 | -140.05 | 45.2 |
| Primary_Somatosensory_Cortex_Forelimb_R  Primary_Somatosensory_Cortex_Dysgranular_R | 9 | -4.461 | 35 | 24.95 | 42.2 |
| Molecular_Layer_of_the_Cerebellum_L | 8 | -4.7136 | -37 | -113.05 | 51.2 |
| Primary_Visual_Cortex_Monocular_Area_L  Primary_Visual_Cortex_Binocular_Area_L | 38 | -5.6486 | -25 | -95.05 | 54.2 |
| Lateral_Secondary_Visual_Cortex_R | 6 | -4.1295 | 65 | -68.05 | 48.2 |
| Olfactory_Bulb_L | 5 | -3.8996 | -4 | 84.95 | 48.2 |
| Primary_Somatosensory_Cortex_Barrel_field_L | 5 | -3.6389 | -55 | -11.05 | 51.2 |
| Primary_Visual_Cortex_Binocular_Area_L | 12 | -4.4264 | -46 | -77.05 | 60.2 |
| Primary_Somatosensory_Cortex_Forelimb_L  Primary_Motor_Cortex_L | 10 | -5.7489 | -40 | 27.95 | 57.2 |
| Secondary_Motor_Cortex_L | 5 | -4.3283 | -13 | 54.95 | 57.2 |
| Primary_Cingular_Cortex_L | 15 | -4.4173 | -4 | 18.95 | 18.95 |
| Retosplenial_Dysgranular_Cortex_R  Retrosplenial_Granular_Cortex_Part_B_R | 5 | -3.7022 | 5 | -50.05 | 63.2 |
| Medial_Parietal_Associative_Cortex_R  Primary_Motor_Cortex_R | 32 | -6.886 | 8 | -35.05 | 63.2 |
| Retosplenial_Dysgranular_Cortex_L | 17 | -4.8079 | -7 | -20.05 | 63.2 |
| Secondary_Motor_Cortex_R  Primary_Motor_Cortex_R | 7 | -4.6126 | 14 | -14.05 | 63.2 |
| Secondary_Motor_Cortex_R | 11 | -5.0861 | 11 | 36.95 | 63.2 |
| Medial_Parietal_Associative_Cortex_R  Lateral_Parietal_Associative_Cortex_R | 7 | -3.7135 | 23 | -32.05 | 66.2 |
| Medio_Lateral_Secondary_Visual_Cortex_L  Medio_Medial_Secondary_Visual_Cortex_L | 13 | -4.9342 | -22 | -53.05 | 69.2 |

Notes: ALFF, amplitude of low-frequency fluctuation; SHR, model group; WKY, normal control group; L, left; R, right. *p* < 0.005, uncorrected, Cluster > 5.

**Table S2. Brain regions with decreased ReHo values in SHR group compared to that in WKY group**

| Negative brain regions | Voxel size | *t*-value | Peak MNI coordinate (mm) | | |
| --- | --- | --- | --- | --- | --- |
|  |  |  | **X** | **Y** | **Z** |
| Hypothalamic_Region_L | 5 | -4.1802 | -10 | -26.05 | -32.8 |
| Basal_Forebrain_Region_L  Optic_Tract_and_Optic_Chiasm_L  Optic_Pathways__L | 57 | -5.4122 | -10 | -5.05 | -20.8 |
| Brainstem_R | 12 | -4.2465 | 20 | -128.05 | -26.8 |
| Hypothalamic_Region_R | 5 | -3.8832 | 11 | -17.05 | -23.8 |
| Basal_Forebrain_Region_R  Optic_Tract_and_Optic_Chiasm_R | 5 | -4.0046 | 11 | 15.95 | -23.8 |
| Entorhinal_Cortex_L  Amygdalopiriform_Cortex_L  Perirhinal_Cortex_L | 23 | -6.3441 | -58 | -32.05 | -8.8 |
| Entorhinal_Cortex_R  Basal_Forebrain_Region_R | 12 | -7.0194 | 44 | -29.05 | -5.8 |
| Basal_Forebrain_Region_L | 5 | -5.1182 | -16 | 15.95 | -20.8 |
| Brainstem_L | 5 | -4.3769 | -19 | -137.05 | -11.8 |
| Brainstem_R  Periventricular_Grey_R  Molecular_Layer_of_the_Cerebellum_R | 7 | -4.0246 | 11 | -140.05 | -2.800003 |
| Dentate_Gyrus_R  Parasubiculum_R  Perirhinal_Area_35_R | 14 | -3.9647 | 41 | -59.05 | 3.2 |
| Perirhinal_Cortex_R  Posterior_Agralunar_Insular_Cortex_R  Lateral_Entorhinal_Cortex_R | 5 | -4.0223 | 59 | -29.05 | -2.8 |
| Cornu_Ammonis_3_L | 7 | -3.8081 | -43 | -47.05 | 0.199997 |
| Basal_Forebrain_Region_L  Basal_Forebrain_Region_R | 5 | -4.0708 | 2 | 33.95 | -2.8 |
| Striatum_R  Corpus_Callosum_and_Associated_Subcortical_White_Matter_R  Fimbria_of_the_Hippocampus3_R | 103 | -6.7069 | 56 | -23.05 | 9.2 |
| Dysgranular_Insular_Cortex_L  Lateral_Primary_Auditory_Cortex_L  Posterior_Agralunar_Insular_Cortex_L | 10 | -6.1032 | -64 | -11.05 | 3.2 |
| Periaqueductal_Gray_R | 7 | -4.0579 | 2 | -59.05 | 9.2 |
| Descending_Corticofugal_Pathways_and_Globus_Pallidum_L  Corpus_Callosum_and_Associated_Subcortical_White_Matter_L  Striatum_L | 56 | -6.2959 | -40 | -32.05 | 21.2 |
| Subiculum_L  Lateral_Entorhinal_Cortex_L  Corpus_Callosum_and_Associated_Subcortical_White_Matter_L | 14 | -5.0545 | -55 | -71.05 | 9.2 |
| PreLimbic_Cortex_L  Olfactory_Bulb_L | 7 | -6.3227 | -31 | 45.95 | 12.2 |
| Olfactory_Bulb_L | 7 | -3.9078 | -28 | 54.95 | 9.2 |
| Thalamus__R  Fimbria_of_the_Hippocampus_R  Cornu_Ammonis_3_R | 38 | -8.061 | 38 | -32.05 | 27.2 |
| Cornu_Ammonis_1_R  Dentate_Gyrus_R | 22 | -7.2776 | 47 | -47.05 | 21.2 |
| Corpus_Callosum_and_Associated_Subcortical_White_Matter_R  PreLimbic_Cortex_R  Primary_Cingular_Cortex_R | 158 | -6.7197 | 2 | 18.95 | 21.2 |
| PreLimbic_Cortex_L  Olfactory_Bulb_L | 6 | -4.0511 | -22 | 39.95 | 21.2 |
| PreLimbic_Cortex_L  Corpus_Callosum_and_Associated_Subcortical_White_Matter_L | 5 | -4.7014 | -13 | 27.95 | 18.2 |
| Dysgranular_Insular_Cortex_L  Agranular_Insular_Cortex_L  Lateral_Primary_Auditory_Cortex_L | 5 | -4.575 | -40 | 30.95 | 24.2 |
| Corpus_Callosum_and_Associated_Subcortical_White_Matter_L  Primary_Somatosensory_Cortex_Barrel_field_L  Primary_Somatosensory_Cortex_Upperlips_L | 6 | -3.6451 | -52 | -11.05 | 24.2 |
| Descending_Corticofugal_Pathways_and_Globus_Pallidum_L | 6 | -5.1706 | -28 | -5.05 | 24.2 |
| Striatum_R | 5 | -4.6285 | 20 | 15.95 | 21.2 |
| Striatum_L | 5 | -5.9277 | -19 | 21.95 | 24.2 |
| Agranular_Insular_Cortex_L  Primary_Somatosensory_Cortex_Jaw_L  Primary_Somatosensory_Cortex_Dysgranular_L | 8 | -3.7496 | -31 | 36.95 | 33.2 |
| Primary_Somatosensory_Cortex_Dysgranular_Zone_R  Primary_Somatosensory_Cortex_Upperlips_R | 7 | -3.917 | 44 | 18.95 | 30.2 |
| Secondary_Motor_Cortex_R  Olfactory_Bulb_R | 8 | -4.0337 | 11 | 57.95 | 30.2 |
| Secondary_Motor_Cortex_L  Olfactory_Bulb_L  PreLimbic_Cortex_L | 10 | -5.3367 | -4 | 60.95 | 36.2 |
| Olfactory_Bulb_L  Glomerular_Layer_of_the_Olfactory_Bulb_L | 9 | -4.8249 | -4 | 81.95 | 36.2 |
| Cornu_Ammonis_1_L  Corpus_Callosum_and_Associated_Subcortical_White_Matter_L  Subiculum_L | 9 | -4.8688 | -46 | -71.05 | 36.2 |
| Dentate_Gyrus_L | 9 | -6.2605 | -28 | -44.05 | 33.2 |
| Corpus_Callosum_and_Associated_Subcortical_White_Matter_L  Primary_Somatosensory_Cortex_Forelimb_L  Striatum_L | 50 | -9.7325 | -34 | -5.05 | 39.2 |
| Molecular_Layer_of_the_Cerebellum_L | 5 | -4.1907 | -46 | -98.05 | 39.2 |
| Corpus_Callosum_and_Associated_Subcortical_White_Matter_L  Secondary_Cingular_Cortex_L | 5 | -3.9076 | -13 | 12.95 | 39.2 |
| Primary_Somatosensory_Cortex_Barrel_field_L  Corpus_Callosum_and_Associated_Subcortical_White_Matter_L  Primary_Somatosensory_Cortex_Hindlimb_L | 17 | -4.6895 | -43 | -14.05 | 42.2 |
| Primary_Motor_Cortex_R  Corpus_Callosum_and_Associated_Subcortical_White_Matter_R | 5 | -3.566 | 23 | -2.05 | 48.2 |
| Primary_Motor_Cortex_R | 8 | -4.6277 | 23 | 15.95 | 42.2 |
| Olfactory_Bulb_R | 11 | -3.9422 | 5 | 87.95 | 48.2 |
| Primary_Visual_Cortex_Binocular_Area_L | 7 | -3.9838 | -40 | -74.05 | 57.2 |
| Primary_Motor_Cortex_L  Secondary_Motor_Cortex_L | 6 | -5.039 | -16 | 24.95 | 54.2 |

Notes: ReHo, regional homogeneity; SHR, model group; WKY, normal control group; L, left; R, right. *p* < 0.005, uncorrected, Cluster > 5.

**Table S3. Brain regions with increased ALFF values in EA group compared to that in SHR group**

| Positive brain regions | Voxel size | *t*-value | Peak MNI coordinate (mm) | | |
| --- | --- | --- | --- | --- | --- |
|  |  |  | **X** | **Y** | **Z** |
| Fornix_L  Optic_Pathways__L | 9 | 5.421 | -16 | -20.05 | -14.8 |
| Brainstem_R  Medial_Lemniscus_R  Hypothalamic_Region_R | 14 | 4.7799 | 20 | -47.05 | 0.199997 |
| Entorhinal_Cortex_R  Amygdalopiriform_Cortex_R | 5 | 3.6708 | 53 | -38.05 | -14.8 |
| Basal_Forebrain_Region_R | 6 | 3.4507 | 20 | 3.95001 | -14.8 |
| Entorhinal_Cortex_R  Lateral_Entorhinal_Cortex_external_part_R  Perirhinal_Cortex_R | 5 | 3.7421 | 56 | -32.05 | -8.8 |
| Molecular_Layer_of_the_Cerebellum_L | 5 | 4.065 | -70 | -116.05 | -5.800003 |
| Thalamus__R | 7 | 4.0062 | 14 | -29.05 | -2.8 |
| Lateral_Entorhinal_Cortex_Internal_part_R  Lateral_Entorhinal_Cortex_R | 5 | 6.317 | 71 | -59.05 | -2.8 |
| Ectorhinal_Cortex_L  Secondary_Auditory_Cortex_Ventral_Part_L | 5 | 5.5302 | -76 | -32.05 | 3.2 |
| Descending_Corticofugal_Pathways_and_Globus_Pallidum_L  Basal_Forebrain_Region_L | 5 | 4.1212 | -25 | -2.04999 | 6.2 |
| Lateral_Primary_Auditory_Cortex_L  Dysgranular_Insular_Cortex_L  Secondary_Somatosensory_Cortex_L | 11 | 7.0799 | -64 | -5.04999 | 6.2 |
| Olfactory_Bulb_L | 6 | 4.6934 | -25 | 33.95 | 6.2 |
| Molecular_Layer_of_the_Cerebellum_L | 5 | 4.4259 | -43 | -131.05 | 12.2 |
| Primary_Auditory_Cortex_R  PreLimbic_Cortex_R  Primary_Somatosensory_Cortex_Upperlips_R | 13 | 5.8672 | 62 | -29.05 | 18.2 |
| Olfactory_Bulb_L | 5 | 4.9528 | -10 | 60.95 | 30.2 |
| Molecular_Layer_of_the_Cerebellum_L  Granule_Cell_Level_of_the_Cerebellum_L | 7 | 4.0051 | -25 | -125.05 | 42.2 |
| Primary_Somatosensory_Cortex_Barrel_field_R | 6 | 4.0566 | 65 | -5.04999 | 42.2 |
| Olfactory_Bulb_R | 7 | 3.7895 | 8 | 78.95 | 39.2 |
| Molecular_Layer_of_the_Cerebellum_R | 6 | 4.6567 | 32 | -131.05 | 42.2 |
| Primary_Visual_Cortex_Binocular_Area_L  Primary_Visual_Cortex_Monocular_Area_L | 11 | 4.9487 | -37 | -89.05 | 54.2 |
| Primary_Motor_Cortex_R | 13 | 7.9169 | 26 | 27.95 | 57.2 |

Notes: ALFF, amplitude of low-frequecy fluctuation; EA, electroacupuncture group; SHR, model group; L, left; R, right; *p* < 0.005, uncorrected, Cluster > 5.

**Table S4. Brain regions with increased ALFF values in MA group compared to that in SHR group**

| Positive brain regions | Voxel size | *t*-value | Peak MNI coordinate (mm) | | |
| --- | --- | --- | --- | --- | --- |
|  |  |  | **X** | **Y** | **Z** |
| Olfactory_Bulb_R | 5 | 3.766 | 29 | 30.95 | -8.8 |
| Secondary_Auditory_Cortex_Ventral_Part_R  Entorhinal_Cortex_R | 5 | 4.3794 | 62 | -32.05 | 3.2 |
| Descending_Corticofugal_Pathways_and_Globus_Pallidum_L | 5 | 6.038 | -34 | -17.05 | 3.2 |
| Molecular_Layer_of_the_Cerebellum_L  Granule_Cell_Level_of_the_Cerebellum_L | 6 | 4.7356 | -22 | -95.05 | 18.2 |
| Striatum_R  Corpus_Callosum_and_Associated_Subcortical_White_Matter_R  Hypothalamic_Region_R | 8 | 5.2354 | 32 | 24.95 | 18.2 |
| Glomerular_Layer_of_the_Olfactory_Bulb_L  Olfactory_Bulb_L | 5 | 4.3486 | -4 | 96.95 | 24.2 |
| Primary_Somatosensory_Cortex_Jaw_L  Primary_Somatosensory_Cortex_Dysgranular_Zone__L | 7 | 4.7816 | -46 | 30.95 | 30.2 |
| Molecular_Layer_of_the_Cerebellum_L  Perirhinal_Cortex_L  Ectorhinal_Cortex_L | 9 | 4.0714 | -49 | -98.05 | 36.2 |
| Granule_Cell_Level_of_the_Cerebellum_L | 5 | 5.2121 | -31 | -116.05 | 39.2 |
| Frontal_Association_Cortex_L | 11 | 6.0107 | -1 | 63.95 | 39.2 |
| Molecular_Layer_of_the_Cerebellum_L | 10 | 4.293 | -40 | -128.05 | 45.2 |
| Primary_Cingular_Cortex_R | 5 | 4.7294 | 1 | 6.95001 | 54.2 |

Notes: ALFF, amplitude of low-frequency fluctuation; MA, manual acupuncture group; SHR, model group; L, left; R, right; *p* < 0.005, uncorrected, Cluster > 5.

**Table S5. Brain regions with increased ReHo values in EA group compared to that in SHR group**

| Positive brain regions | Voxel size | *t*-value | Peak MNI coordinate (mm) | | |
| --- | --- | --- | --- | --- | --- |
|  |  |  | **X** | **Y** | **Z** |
| Basal_Forebrain_Region_R | 7 | 4.7691 | 23 | -2.05 | -29.8 |
| Brainstem_R | 8 | 5.3912 | 17 | -128.05 | -23.8 |
| Entorhinal_Cortex_R | 6 | 3.3653 | 32 | -8.05 | -23.8 |
| Brainstem_L  Middle_Cerebellar_Peduncle__L  Inferior_Olive_n_Pyramidal Decusation_n_SP5_L | 5 | 3.7012 | -25 | -74.05 | -17.8 |
| Optic_Pathways__R  Fornix_R | 5 | 3.8479 | 14 | -20.05 | -17.8 |
| Molecular_Layer_of_the_Cerebellum_R  Granule_Cell_Level_of_the_Cerebellum_R | 5 | 4.6703 | 74 | -116.05 | -2.800003 |
| Brainstem_L  Medial_Lemniscus_L | 5 | 4.2382 | -19 | -47.05 | 0.199997 |
| Thalamus__L | 5 | 4.0637 | -19 | -29.05 | -2.8 |
| Striatum_R  Olfactory_Bulb_R | 8 | 5.66 | 20 | 30.95 | 0.199997 |
| Brainstem_R | 5 | 3.3812 | 8 | -53.05 | 0.199997 |
| Descending_Corticofugal_Pathways_and_Globus_Pallidum_R | 5 | 4.7685 | 35 | -14.05 | 9.2 |
| Hypothalamic_Region_R  Corpus_Callosum_and_Associated_Subcortical_White_Matter_R | 11 | 4.4459 | 53 | -23.05 | 6.2 |
| Agranular_Insular_Cortex_R  Dysgranular_Insular_Cortex_R | 6 | 3.965 | 47 | 30.95 | 9.2 |
| Dysgranular_Insular_Cortex_R  Lateral_Primary_Auditory_Cortex_R  Primary_Somatosensory_Cortex_Upperlips_R | 7 | 4.6063 | 59 | 27.95 | 12.2 |
| Striatum_R | 6 | 4.0965 | 32 | 15.95 | 12.2 |
| Primary_Auditory_Cortex_L  Primary_Somatosensory_Cortex_Upperlips_L  Secondary_Auditory_Cortex_Dorsal_Part_L | 5 | 4.0757 | -61 | -26.05 | 18.2 |
| PreLimbic_Cortex_R  Basal_Forebrain_Region_R | 5 | 4.5639 | 8 | 21.95 | 24.2 |
| Olfactory_Bulb_R | 6 | 4.3184 | 26 | 75.95 | 33.2 |
| Molecular_Layer_of_the_Cerebellum_L | 5 | 5.1518 | -49 | -131.05 | 39.2 |
| PreLimbic_Cortex_R | 5 | 5.1376 | 2 | 42.95 | 33.2 |
| Molecular_Layer_of_the_Cerebellum_R | 6 | 3.5676 | 17 | -116.05 | 42.2 |
| Lateral_Parietal_Associative_Cortex_L | 7 | 4.6557 | -46 | -35.05 | 66.2 |

Notes: ReHo, regional homogeneity; EA, electroacupuncture group; SHR, model group; L, left; R, right; *p* < 0.005, uncorrected, Cluster > 5.

**Table S6. Brain regions with increased ReHo values in MA group compared to that in SHR group**

| Positive brain regions | Voxel size | *t*-value | Peak MNI coordinate (mm) | | |
| --- | --- | --- | --- | --- | --- |
|  |  |  | **X** | **Y** | **Z** |
| Basal_Forebrain_Region_R  Entorhinal_Cortex_R | 7 | 4.2082 | 41 | -5.05 | -11.8 |
| Substantia_Nigra_R  Brainstem_R | 6 | 4.5707 | 20 | -56.05 | -5.8 |
| Primary_Auditory_Cortex_L  Hypothalamic_Region_R  Ectorhinal_Cortex_L | 11 | 4.2562 | -61 | -41.05 | 9.2 |
| Striatum_R  Corpus_Callosum_and_Associated_Subcortical_White_Matter_R  Primary_Auditory_Cortex_R | 10 | 4.9278 | 56 | -26.05 | 9.2 |
| PreLimbic_System_L  Olfactory_Bulb_L | 6 | 3.9693 | -31 | 45.95 | 12.2 |
| Corpus_Callosum_and_Associated_Subcortical_White_Matter_L  Striatum_L  Dysgranular_Insular_Cortex_L | 9 | 4.6709 | -31 | 24.95 | 18.2 |
| Primary_Somatosensory_Cortex_Barrel_field_R | 5 | 4.4029 | 56 | -11.05 | 27.2 |
| Olfactory_Bulb_R | 5 | 3.6908 | 8 | 87.95 | 39.2 |
| Retrosplenial_Granular_Cortex_Part_B_R  Corpus_Callosum_and_Associated_Subcortical_White_Matter_R | 5 | 4.6879 | 2 | -23.05 | 48.2 |
| Primary_Cingular_Cortex_R  Secondary_Motor_Cortex_R | 13 | 5.3026 | 8 | 9.95 | 51.2 |

Notes: ReHo, regional homogeneity; MA, manual acupuncture group; SHR, model group; L, left; R, right; *p* < 0.005, uncorrected, Cluster > 5.

**Table S7. The decreased strengths of the FC in SHR group compared to that in WKY group with the HHA.R as the seed**

| Brain regions | Voxel size | *t*-value | Peak MNI coordinate (mm) | | |
| --- | --- | --- | --- | --- | --- |
|  |  |  | **X** | **Y** | **Z** |
| Brainstem_R | 3 | -3.5002 | 5 | -107.05 | -23.8 |
| Entorhinal_Cortex_L | 2 | -3.4848 | -46 | -26.05 | -23.8 |
| Basal_Forebrain_Region_R | 4 | -3.765 | 14 | 24.95 | -14.8 |
| Basal_Forebrain_Region_R | 2 | -4.0368 | 26 | -11.05 | -11.8 |
| Brainstem_L | 3 | -4.2152 | -22 | -41.05 | -8.8 |
| Brainstem_R | 4 | -4.4487 | 8 | -119.05 | -5.800003 |
| Granule_Cell_Level_of_the_Cerebellum_L | 2 | -3.6025 | -34 | -104.05 | 12.2 |
| Perirhinal_Area_36_L | 2 | -3.4888 | -49 | -77.05 | 15.2 |
| Molecular_Layer_of_the_Cerebellum_R | 2 | -4.6536 | 53 | -101.05 | 18.2 |
| Molecular_Layer_of_the_Cerebellum_R | 2 | -3.9077 | 38 | -92.05 | 27.2 |
| Molecular_Layer_of_the_Cerebellum_L | 2 | -4.0832 | -16 | -143.05 | 33.2 |
| Granule_Cell_Level_of_the_Cerebellum_L  Molecular_Layer_of_the_Cerebellum_L | 2 | -3.3452 | -7 | -110.05 | 33.2 |
| Deeper_Layers_of_the_Superior_Colliculus_L | 2 | -3.6923 | -25 | -86.05 | 33.2 |
| Molecular_Layer_of_the_Cerebellum_L | 2 | -3.6531 | -40 | -134.05 | 36.2 |
| Molecular_Layer_of_the_Cerebellum_R | 2 | -3.1506 | 23 | -98.05 | 48.2 |
| Primary_Somatosensory_Cortex_Barrel_field_L | 2 | -3.3899 | -52 | -23.05 | 45.2 |
| Secondary_Motor_Cortex_R | 2 | -4.1027 | 11 | 45.95 | 54.2 |

Notes: FC, functional connectivity; SHR, model group; WKY, normal control group; HHA.R, hypothalamic region.R; L, left; R, right. *p* < 0.005, uncorrected, Cluster > 2.

**Table S8. The decreased strengths of the FC in SHR group compared to that in WKY group with the Ent.R as the seed**

| Brain regions | Voxel size | *t*-value | Peak MNI coordinate (mm) | | |
| --- | --- | --- | --- | --- | --- |
|  |  |  | **X** | **Y** | **Z** |
| Optic_Pathways__R  Optic_Tract_and_Optic_Chiasm_R | 2 | -4.273 | 17 | -17.05 | -23.8 |
| Hypothalamic_Region_R | 2 | -3.1137 | 14 | -38.05 | -20.8 |
| Brainstem_R | 2 | -3.4393 | 5 | -122.05 | -11.8 |
| Brainstem_L | 2 | -4.4773 | -25 | -122.05 | -11.8 |
| Brainstem_L | 3 | -5.4705 | -25 | -149.05 | -5.800003 |
| Brainstem_L | 2 | -3.4427 | -19 | -41.05 | -8.8 |
| Posterior_Agralunar_Insular_Cortex_L | 2 | -3.8541 | -61 | -5.05 | -2.8 |
| Brainstem_L  Medial_Lemniscus_L | 4 | -3.7584 | -16 | -47.05 | 0.199997 |
| Ectorhinal_Cortex_L | 2 | -3.5719 | -73 | -35.05 | 0.199997 |
| Granule_Cell_Level_of_the_Cerebellum_L | 2 | -4.1009 | -64 | -113.05 | 6.199997 |
| Granule_Cell_Level_of_the_Cerebellum_R | 3 | -4.7942 | 20 | -98.05 | 12.2 |
| Granule_Cell_Level_of_the_Cerebellum_R  Molecular_Layer_of_the_Cerebellum_R | 4 | -3.3103 | 38 | -101.05 | 12.2 |
| Ectorhinal_Cortex_R  Lateral_Entorhinal_Cortex_R | 2 | -3.5862 | 74 | -74.05 | 9.2 |
| Striatum_R | 2 | -3.2977 | 47 | 0.949997 | 12.2 |
| Secondary_Somatosensory_Cortex_R | 2 | -4.6818 | 71 | -2.05 | 18.2 |
| Striatum_R | 2 | -3.4838 | 32 | 15.95 | 21.2 |
| Molecular_Layer_of_the_Cerebellum_R | 2 | -3.4739 | 11 | -134.05 | 27.2 |
| Molecular_Layer_of_the_Cerebellum_L | 2 | -4.1242 | -34 | -119.05 | 27.2 |
| Molecular_Layer_of_the_Cerebellum_R  Perirhinal_Area_36_R | 2 | -3.1908 | 41 | -92.05 | 27.2 |
| Deeper_Layers_of_the_Superior_Colliculus_R | 2 | -4.188 | 20 | -74.05 | 27.2 |
| Dentate_Gyrus_R  Ventral_Hippocampal_Commissure_L | 2 | -3.0272 | 2 | -14.05 | 27.2 |
| Temporal_Associative_Cortex_R | 3 | -4.6056 | 65 | -77.05 | 30.2 |
| Dentate_Gyrus_L | 2 | -3.9557 | -13 | -26.05 | 30.2 |
| Cornu_Ammonis_1_R  Dentate_Gyrus_R | 2 | -3.1878 | 11 | -23.05 | 30.2 |
| PreLimbic_Cortex_R | 2 | -3.7246 | 8 | 36.95 | 30.2 |
| Molecular_Layer_of_the_Cerebellum_R | 2 | -4.0952 | 14 | -134.05 | 33.2 |
| Primary_Motor_Cortex_R  Primary_Somatosensory_Cortex_Dysgranular_R  Primary_Somatosensory_Cortex_Forelimb_R | 3 | -3.6043 | 29 | 15.95 | 39.2 |
| Retrosplenial_Granular_Cortex_Part_A_L | 3 | -3.3869 | -16 | -65.05 | 45.2 |
| Lateral_Secondary_Visual_Cortex_L | 3 | -4.672 | -64 | -68.05 | 51.2 |
| Primary_Somatosensory_Cortex_Barrel_field_L | 2 | -3.2652 | -55 | 0.949997 | 51.2 |
| Primary_Somatosensory_Cortex_Dysgranular_L | 2 | -4.6367 | -52 | 6.95 | 48.2 |
| Primary_Somatosensory_Cortex_Forelimb_R  Primary_Somatosensory_Cortex_Jaw_R | 6 | -4.3491 | 41 | 30.95 | 51.2 |
| Corpus_Callosum_and_Associated_Subcortical_White_Matter_R | 2 | -3.8305 | 26 | -47.05 | 51.2 |
| Retosplenial_Dysgranular_Cortex_R  Retrosplenial_Granular_Cortex_Part_B_R | 2 | -4.2129 | 2 | -23.05 | 51.2 |
| Molecular_Layer_of_the_Cerebellum_R | 2 | -3.6266 | 5 | -134.05 | 54.2 |
| Retosplenial_Dysgranular_Cortex_R | 2 | -3.0743 | 8 | -62.05 | 66.2 |
| Medio_Lateral_Secondary_Visual_Cortex_L | 2 | -3.8532 | -31 | -44.05 | 63.2 |

Notes: FC, functional connectivity; SHR, model group; WKY, normal control group; Ent.R, entorhinal cortex.R; L, left; R, right. *p* < 0.005, uncorrected, Cluster > 2.
